# The role of evidence, context, and facilitation in an implementation trial: implications for the development of the PARIHS framework

**DOI:** 10.1186/1748-5908-8-28

**Published:** 2013-03-09

**Authors:** Jo Rycroft-Malone, Kate Seers, Jackie Chandler, Claire A Hawkes, Nicola Crichton, Claire Allen, Ian Bullock, Leo Strunin

**Affiliations:** 1 School of Healthcare Sciences, Bangor University, Ffriddoedd Road, Bangor, UK; 2 RCN Research Institute, School of Health and Social Studies, Warwick University, Coventry, UK; 3 Cochrane Collaboration, Summertown Pavilion, Oxford, UK; 4 Faculty of Health and Social Care, London South Bank University, London, UK; 5 National Clinical Guideline Centre (NCGC), Royal College of Physicians, London, UK; 6 Former President of the Royal College of Anaesthetists, London, UK

## Abstract

**Background:**

The case has been made for more and better theory-informed process evaluations within trials in an effort to facilitate insightful understandings of how interventions work. In this paper, we provide an explanation of implementation processes from one of the first national implementation research randomized controlled trials with embedded process evaluation conducted within acute care, and a proposed extension to the Promoting Action on Research Implementation in Health Services (PARIHS) framework.

**Methods:**

The PARIHS framework was prospectively applied to guide decisions about intervention design, data collection, and analysis processes in a trial focussed on reducing peri-operative fasting times. In order to capture a holistic picture of implementation processes, the same data were collected across 19 participating hospitals irrespective of allocation to intervention. This paper reports on findings from data collected from a purposive sample of 151 staff and patients pre- and post-intervention. Data were analysed using content analysis within, and then across data sets.

**Results:**

A robust and uncontested evidence base was a necessary, but not sufficient condition for practice change, in that individual staff and patient responses such as caution influenced decision making. The implementation context was challenging, in which individuals and teams were bounded by professional issues, communication challenges, power and a lack of clarity for the authority and responsibility for practice change. Progress was made in sites where processes were aligned with existing initiatives. Additionally, facilitators reported engaging in many intervention implementation activities, some of which result in practice changes, but not significant improvements to outcomes.

**Conclusions:**

This study provided an opportunity for reflection on the comprehensiveness of the PARIHS framework. Consistent with the underlying tenant of PARIHS, a multi-faceted and dynamic story of implementation was evident. However, the prominent role that individuals played as part of the interaction between evidence and context is not currently explicit within the framework. We propose that successful implementation of evidence into practice is a planned facilitated process involving an interplay between individuals, evidence, and context to promote evidence-informed practice. This proposal will enhance the potential of the PARIHS framework for explanation, and ensure theoretical development both informs and responds to the evidence base for implementation.

**Trial registration:**

ISRCTN18046709 - Peri-operative Implementation Study Evaluation (PoISE).

## Background

Implementation research is ‘the scientific study of methods to promote the systematic uptake of clinical research findings and other evidence-based practice into routine practice, and hence improve the quality…of healthcare’ [1]. Historically, there had been a lack of attention to theory in implementation research, however in recent years there has been a growing interest in its development and use [2-8]. Theory is relevant and applicable to implementation research in a number of ways [3,7,9-11], including in the choice and development of interventions, for identifying appropriate outcomes, measures, and variables of interest, and in guiding the evaluation of implementation processes and outcomes. Despite an increasing attention to theory in implementation research, there is still much to learn about theory use and development. In this paper, we report on the findings from a theoretically grounded process evaluation that was embedded in a large trial evaluating implementation interventions [12]. The aim is to provide an explanation of implementation processes, and how this contributes to theory building through the development of the Promoting Action on Research Implementation in Health Services (PARIHS) framework [13-15].

PARIHS was embedded within our evaluation framework because it provided a potentially useful heuristic to guide the development of the study and evaluate the process of implementation. PARIHS represents the complexity of implementation by considering the three core elements and their sub-elements to be dynamic and interrelated. Successful implementation of research evidence is conceived as a function of evidence, context, and facilitation. Specifically, the working proposition is that the most successful implementation will occur when evidence is robust and practitioners ‘agree’ with it, the context is receptive, and where implementation processes are appropriately facilitated by internal and/or external facilitators [13,14].

PARIHS is an example of the development of a middle range explanation or theory about implementation [16]. As such, it does not offer absolute prediction or explain all ‘observable uniformities of social behavior’ [16], but provides a conceptual framework that organizes various important components or influences that combine and interact in more or less uniform ways or patterns. While PARIHS explains some of the interactions between evidence, context, and facilitation, the pattern of interactions and related outcomes will be contingent upon implementation settings. Therefore while a degree of regularity or patterns can be observed across time and place, middle-range theory/ies are also in constant need of better specification in order to increase their explanatory ‘power.’

PARIHS appears to have good face validity in that it has been often used in implementation activity and is widely cited in the international literature. However, in their review of the use of PARIHS, Helfrich et al. [17] found that others had tended to use the framework retrospectively. They argue that in order to move the framework forward researchers need to prospectively use PARIHS to design and evaluate implementation research and projects. The authors also make a general point that researchers are not conducting prospective implementation studies based on conceptual frameworks, or on the explicit use of theory at all (17: p16).

There are a number of reviews about the effectiveness of interventions to promote the uptake of evidence in practice [18-24]. Generally, this evidence base indicates that there may be more promise in the use of interventions that are theoretically based, interactive, and tailored to barriers [25]. However, because there has been a lack of attention to implementation processes within studies such as those included in these reviews, it is difficult to determine why and how interventions may have worked or not, or how to tailor interventions. The ability to learn from implementation studies would be improved by greater attention on theoretically driven formative and/or process evaluations to help determine how interventions work in different contexts, and to promote the transferability of findings to other settings [10,26].

In order to fully understand implementation processes and impacts within the context of a large pragmatic trial evaluating interventions to improve peri-operative fasting times, we designed a process evaluation in which PARIHS was embedded [12]. The main aims of the process evaluation were to determine how the implementation interventions were received within sites, whether any impacts were observed locally, and how implementation processes played out. Given the gap in the literature with respect to the processes of implementation, the use of theory, and specifically the prospective use of PARIHS, this paper presents an explanation of how the study’s findings provided an opportunity to review this conceptual framework. An extension to enhance PARIHS as a conceptual framework that represents implementation is considered.

## Methods

**Figure 1:**
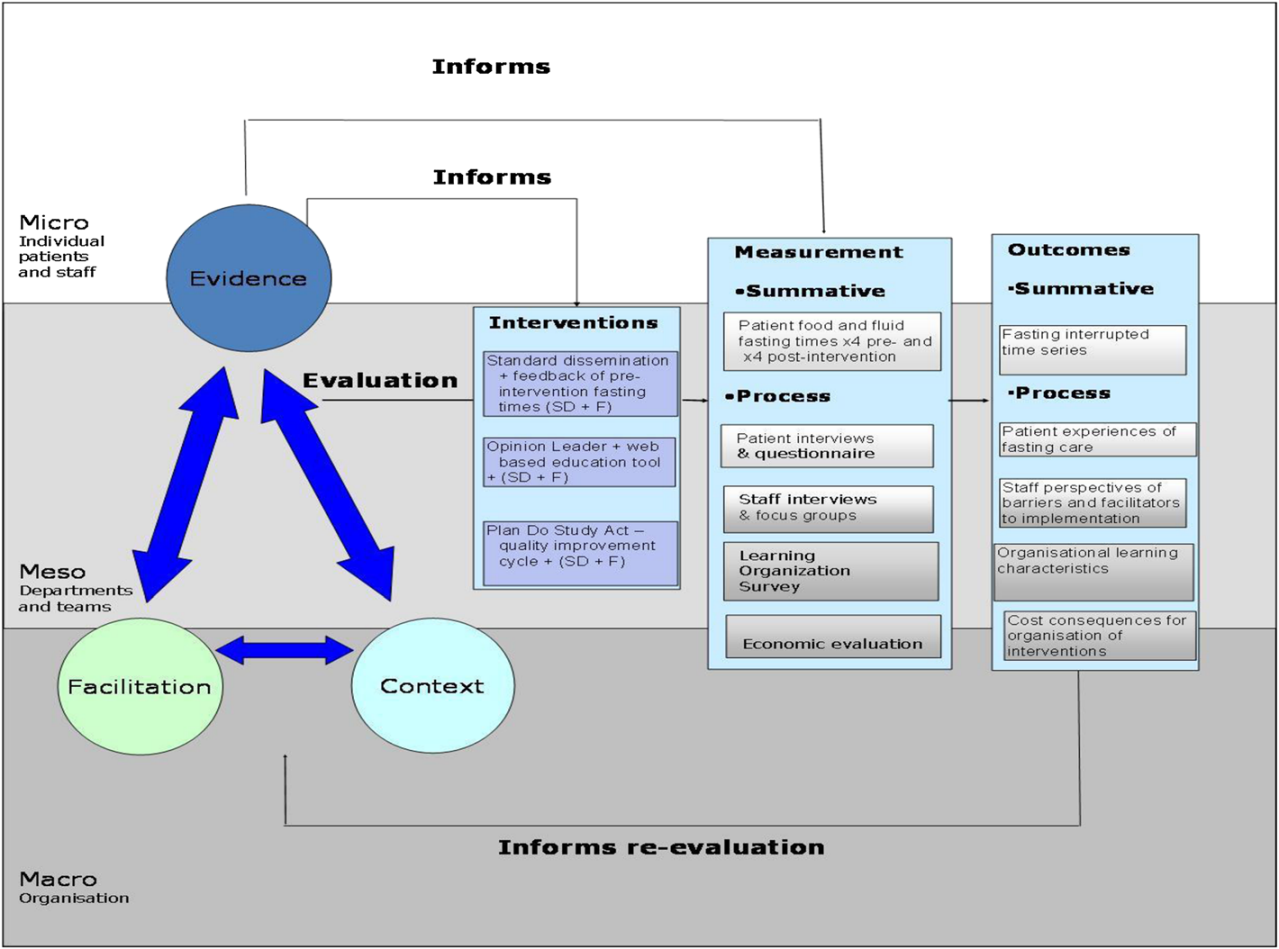
Study Evaluation Framework.

The framework developed for this study reflects the multiple components at play in implementation work (Figure 1). PARIHS is embedded in the framework to represent the potential contribution of the nature and type of evidence, the qualities of the context in which the evidence is being introduced, and the way the process is facilitated. Additionally, the study’s framework incorporates the idea that there are influential factors at micro, meso, and macro organizational layers of context [6,27]. The framework also represents implementation as a process and outcome that is more or less influenced by the interventions and the context in which they are being introduced. We did not set out to test the framework per se, but used it to guide decisions about intervention design, data collection (focus and content), and analysis processes.

### Interventions

Strategies for the implementation of two of the evidence-based guideline recommendations were designed. The recommendations include that patients could have water up to two hours and food up to six hours before induction of anaesthesia, with resumption of fluids when fully awake [28]. Drawing on a variable evidence base about the relative effectiveness of implementation interventions [18-24], three trial interventions were developed and randomly allocated to 19 participating hospitals in England, Scotland, Wales, and Northern Ireland: standard dissemination, a web-based resource championed by an opinion leader(s), and a Plan-Do-Study-Act (PDSA) intervention. Table 1 shows the characteristics of each intervention, their intended mechanism(s) of action, and link to PARIHS elements.

**Table 1:** Intervention characteristics

	**Intervention**		
	**Standard Dissemination (SD)**	**SD+ Web**-**based resource and opinion leader(s)**	**SD + PDSA**
**Source**	Guideline package, including RCN/RCoA national guidelines, patient version, PowerPoint presentation about implementation	Guideline package and web-based learning resource	Guideline package and adaptation of the improvement cycles from the former Modernisation Agency Improvement Leaders Guide. Include readiness to change tool.
**Format**	Paper and CD	Computer	PDSA paper-based package and facilitator led
**Target**	Trust/local health board – those who usually received national guideline information	Multi-professional staff, individuals and/or groups	Multi-professional staff groups
**Delivered by**	Unknown	Local opinion leader(s)	Local PDSA facilitator
**Duration**	Six months	Six months	Six months
**Number of events**	One	Multiple, but not specified	Six meetings specified plus local audit activity
**Proximity to practice**	Remote	Arms length with front line championing	Front line
**Setting**	Trust	Ward and/or theatre	Ward/theatre/pre-admission clinic
**Link to PARIHS**	Evidence:	Evidence and facilitation:	Evidence, context and facilitation:
**Successful Implementation**(**SI**) = the use of the recommendations in practice with impact on practice and patient outcomes	- research evidence in the guideline (E)	- research evidence in the guideline (E)	- research evidence in the guideline (E)
	- patient guideline (E)	- patient guideline (E)	- patient guideline (E) local evidence in PDSA approach(E)
		- championing, awareness raising, role modelling, other appropriate facilitative activities (F)	- practitioner experience in PDSA approach (E)
			- local evidence particularisation (E,C,F)
			- team working (C) project leader (F)
			- tailoring practice interventions (F andC)
**Intended mechanism of action**	Awareness raising	Awareness raising + Social influence and education	Awareness raising + facilitation and localising to front line practice context

In relation to the study’s evaluation framework, these interventions had the potential to work at multiple levels within an organization’s context and with varying degrees of interactivity from none (standard dissemination) to local particularisation based on an assessment of readiness (PDSA). All included attention to a robust evidence base in the form of a guideline that had been developed using National Institute for Health and Clinical Excellence’s (NICE) approach. There were facilitation components in the web-based resource and opinion leader intervention, as well as in the PDSA intervention, which included a local facilitator. Successful implementation in this study meant the use of the recommendations in practice and impact on practice and patient outcomes. The intervention period was six months.

### Data collection

Data were collected pre- and post-intervention between November 2006 and February 2009 across the 19 participating hospitals [12]. The same data were collected irrespective of allocation to intervention, and participants purposively sampled because of their role within the study: change agent, key contact, and recipients (staff and patients) of the interventions. We interviewed all change agents and key contacts pre- and post-intervention apart from in one site were ill health prevented follow up (see Tables 2 and 3). Ward staff identified potential patients for interview, and key contacts identified ward staff who might be willing to participate in a focus group [12]. In this paper we draw on the following data sources.

**Table 2:** Type and number of participants

	**Pre-intervention**	**Post-intervention**	**Total**
Key Contact Interviews	16	12	28
Change Agent Interviews	12	12	21
Patient Interviews	35	35	70
Focus Groups		5 Total participants = 32 (7, 7, 9, 6, 3)	32 participants

**Table 3:** Individual interview participants

**Pre**-**intervention key contact and change agent interviews**-**N** = **31**	**Post**-**intervention change agent interviews**-**N** = **12**	**Post**-**intervention key contact follow up interviews**-**N** = **12**
19 Key contacts	6 PDSA facilitators	12** interviews from a possible 19
12 Change agents	5 Opinion leaders	
	1 Trust key contact*	

*One site lost a PDSA facilitator due to ill health key contact interviewed.

**One key contact not available so local investigator interviewed.

Semi- structured audio-recorded interviews were conducted with patients from each hospital during pre- and post-intervention data collection periods about their experiences of fasting. An interview schedule was developed with the help of the project’s patient advisor. Ward staff identified potential patients for interview and the majority of patients were interviewed within three days post-operatively.

Semi-structured audio-recorded telephone interviews took place with PDSA facilitators and opinion leaders about their experiences of implementation including activities, barriers and facilitators, and perceived impact were collected in pre- and post-intervention periods.

Semi-structured audio recorded telephone interviews took place with hospital key contacts who had facilitated access and the running of the project locally about their experiences of intervention implementation, including the identification of any changes in practice and use of resources were conducted pre- and post-intervention, including whether these had been sustained three months post-intervention.

The interview topics for patient and staff interviews were developed based on the evaluation framework and therefore included questions about perceptions of the evidence about fasting, the receptiveness of the contexts of implementation, the role and activities of the opinion leaders and PDSA facilitators, and any other facilitators and barriers perceived and experienced by participants.

Multi-professional audio-recorded focus groups were conducted by two moderators to gather information about the experience of staff in clinical areas where the study had been taking place post-intervention. A sample of hospitals was identified within each intervention arm as having made the largest change to fluid fast time (n = 3) and those that had made the smallest/no/marginal change (n = 2). The schedule was based on emerging findings from the staff interview data and included awareness and impact of the study/interventions, changes to fasting practice, inter-professional issues, and processes of practice change, including barriers and facilitators.

### Data analysis and integration

Qualitative audio-recorded data was transcribed in full, and managed in N*DIST 5 (pre-intervention) and NVIVO 7 (post-intervention). A process of inductive and deductive analysis was undertaken informed by Ritchie and Spencer’s [29] approach to analysis, specifically, their approach to concept identification and thematic framework development. First, data were analysed within data set (i.e., focus groups, staff interviews, patient interviews). We coded eight to ten interview transcripts in each data set inductively, and these codes were used to develop an analysis framework. This framework was used to code the remaining interviews, and it was refined as new codes emerged. Second, the findings that emerged within data set were reviewed and mapped against the key elements of the study framework. This resulted in the emergence of higher level themes across the core elements of evidence, context, and facilitation. The analysis process was carried out by three members of the research team, which included cross-checking coding and themes. Emerging themes were also shared periodically with the whole research team as an additional check on credibility.

### Ethics

This study was approved by a multi-site ethics committee (06/MRE01/20).

## Results

Overall findings from the trial are published separately [12]. Participants and sources of data are presented in Tables 2, 3, and 4.

**Table 4:** Focus group participants

**Site ID**	**Intervention Arm**	**Staff groups involved**	**Number of participants**
A	Standard dissemination	Ward sister, ward staff nurses, ward HCA, theatre staff nurse, recovery staff nurse, ODP	7
F	Standard dissemination	Consultant anaesthetist (clinical director), theatre manager, theatre sister, theatre staff nurse, ward sisters/charge nurses, ODPs	9
J	Opinion Leader + Web resource +SD	Ward nurses, theatre and recovery nurses, consultant anaesthetist and SPR anaesthetist	7
S	Opinion Leader + Web resource +SD	Nurses (ward sister and ward staff nurse), consultant anaesthetist	3
N	Plan Do Study Act + SD+	ODP manager, theatre nurses and nurse manager, ward staff nurses	6

The results from the trial showed no significant effect of the interventions on the primary outcome of fluid fasting time. The process evaluation data provides a rich picture of implementation processes that offers an explanation for the trial findings, and about how the implementation of interventions played out within the practice context. Particular themes about implementation facilitators and barriers were not specific to interventions; rather we observed patterns across sites. There are some findings that are specific to the components of particular interventions. The main tenets of PARIHS: evidence, context, and facilitation are used to present the findings. The links to particular interventions and impact(s) are embedded within these descriptions. Consistent with implementation in the reality of the clinical setting, there is a complexity and dynamism that underlies these findings. Linkages and interactions will be highlighted in the following sections, and considered in more detail in the discussion.

### Evidence

The research base for any potential change to practice was strong in that the recommendations were underpinned by robust randomized controlled trials. The message for practice was also ‘simple’ and framed within the guideline as the ‘2 and 6 rule’—patients can drink clear fluids up to two hours, and eat up to six hours, before anaesthesia. Apart from one site who used their existing processes to disseminate the guideline package across relevant parts of the organization, it was unclear what happened to it once it was received by hospital directors in other sites. Therefore, we could surmise that the evidence may not have reached the potential users in most sites. Two main findings emerged relating to evidence and its impact on the potential to change practice.

### Uncontested evidence mediated by caution

Data from interviews with change agents and key contacts who were either senior nursing or anaesthetic staff showed that most respondents believed the evidence base for shortening fasting times to be robust:

‘I think there is good evidence and this is just being reinforced over the last few years…there is a rationality behind it.’ (change agent, site H)

Participants’ perceptions of their colleagues’ views of the evidence base were also, on the whole, positive. Apart from some wanting clarity about how to interpret recommendations, for example, how much water patients could drink pre-operatively; the evidence base was relatively uncontested by those involved in the study. These perceptions did not change pre- and post-intervention.

This positive attitude to the research base was mediated by practitioner and patient judgements about the need for caution, and perceived attitudes to risk taking. The need for managing operating lists meant that individual staff reportedly took a cautionary approach to fasting practice:

‘I think people are fearful that something will go wrong…so they always take what they see as the safest option, which is to fast people from midnight…I think it’s lack of confidence.’ (key contact, site F).

Participants at one focus group raised a question about whether clinicians were perhaps overestimating the risk of aspiration compared with the risks of prolonged fasting. In another focus group, it was reported that the inexact nature of estimating time for each operation meant many anaesthetists erred on the site of caution. In contrast, another anaesthetist stated that she would ‘modify the rules’ rather than postpone or cancel cases.

Some patients also expressed a cautious attitude towards the advice they had been given because they did not want to jeopardize their treatment:

‘…I always think that instructions like that, there’s a good reason…I abide by them religiously…I just thought I’d sooner err on the side of safety.’ (patient, site D)

### Localizing evidence

The guideline recommendations were mainly localized through local policies rather than, for example, consensus processes. At some sites existing policy was in line with the guideline evidence, and in others it was similar (e.g., three hours fluid fast). For some, the fasting policy needed updating and two sites did not have a policy. Change agents and key contacts reported that a number of activities had taken place to embed recommendations into local practice including changing patient information letters and leaflets, and information for staff. While this showed the evidence was used locally, it did not impact on practice [12].

In summary, while the research evidence base underpinning the recommendations being implemented in this study was empirically robust and broadly accepted by practitioners (judged as strong within the PARIHS framework), individuals’ responses to it, including patients’ reactions, varied and/or were mediated by other factors. There was a difference between agreeing with evidence, and using it to make decisions and/or change services. In this study, the interventions implemented did not significantly affect fasting times.

### Context

Across the 19 trusts in the study, some cross-cutting themes emerged from the data about the influence that contextual factors at micro (individual), meso (team within the elective surgery department) and macro (hospital) levels had on implementation processes. These themes interact with each other such that, for example, emotional reactions may be a function of inter-professional issues, and a product of individual, team and organizational communication.

### Micro

#### Emotional response

In addition to cautionary practice, there was also evidence that practitioners were anxious about changing traditional practice; these people were described by one anaesthetist participant as ‘dinosaurs.’ In focus groups several comments were made about clinicians being afraid of moving on with a new practice, and that the amount of work required to change would be too daunting.

Such anxiety and caution seemed to lead to an emotional response, with some staff becoming ‘confrontational’ with those that were trying to instigate changes. For example, one respondent spoke of senior consultant anaesthetists challenging junior anaesthetists attempting to implement the recommendations:

‘…when they have a consultant say “don’t be so ridiculous” and “if you run into trouble don’t ask me to help you” it doesn’t help…’ (anaesthetist opinion leader, site E)

### Meso

#### Inter-professional issues

This finding was a significant issue across all data and includes different professional approaches, leadership, power and hierarchical structures, and professional cultures. Fasting practice was influenced by how the disciplines functioned together, sometimes bringing them into conflict because they had different objectives, ways of working and power bases. One anaesthetist PDSA facilitator commented that the guidelines ‘are embedded in so many different cultures…so many different aspects of the organization’ (site R) that it makes change difficult to use and embed.

### Ownership and decision-making authority

One of the challenges encountered concerned the ‘ownership’ of fasting practice. A need was expressed for establishing clarity about roles and responsibilities and for all team members to take ownership:

‘…I’d like to see all of us taking more ownership and being more proactive in actually thinking about how long the patient’s been fasted for. At the moment I think everybody else thinks it’s everybody else’s job to do it and it doesn’t get done.’ (Senior nurse key contact/change agent, site M).

This was tempered by a concern expressed by some nurses about getting into trouble if patients were not fasted long enough, with which some anaesthetists sympathized:

‘I fully understand where nurses are coming from…if they get it wrong then they get into a lot of difficulty and quite a lot of abuse from irate doctors, because they’ve messed up the theatre list basically…we need a robust mechanism.’ (anaesthetist opinion leader site J)

### Communication

Bringing fasting practice more in line with the guideline recommendations requires a multi-disciplinary team approach and communication within and across teams and departments. Data shows examples of some challenges with communication, which meant that individualising patients’ fasting times in line with recommendations would be difficult, if not impossible to achieve:

‘We’ve had a phone call from theatre to say this patient’s been cancelled and you can feed and water them. Half an hour later we’ve had a phone call saying has she or he been fed and watered, she can go down…so there’s been miscommunication or been told completely wrong.’ (nurse, focus group – site A)

There were also reports of ward-based team communication problems, which had led to prolonged delays or fasting times for patients.

### Macro

#### Commitment and buy in

This study was conducted at a time of major NHS changes, including reconfigurations, changes to junior medical staff training grades, and a re-focus on financial deficits impacting on staff turnover, workforce reviews, and reorganizations. Staff reported feeling overwhelmed by competing priorities, and that this project came ‘at a bad time.’ While hospitals had signed up to being involved in the project, the managerial support for it was variable, and consequently so were the commitment and resources dedicated to it. A number of resource issues hindered implementation effort such as time (specifically for opinion leaders and PDSA facilitators to enact their roles, and for education sessions and team meetings), turnover of staff, and lack of permanent staff. The success of the project locally was vested in individual’s enthusiasm and commitment:

‘…it came from me, I would say…the clinical director knows about it and he’s happy to let me do that…the wards do, but they don’t care about the nuts and bolts of it.’ (anaesthetist key contact – site C)

Apart from at one site in which the nursing director was supportive and prioritized the issue (a site that was allocated to standard dissemination and did make improvements) generally at hospital level, the buy-in to evidence-based peri-operative fasting was not evident. This finding is likely to be fairly typical of many clinical issues underpinned by national guidelines and a function of how organizations set their priorities.

### Different starting points

One of the critical issues that limited the potential to make significant changes to fasting times within the intervention period was that hospitals were at different starting points with respect to fasting practice and preparedness. Some hospitals did not have a written fasting policy and had to develop one during the intervention phase, which left limited time to implement it. In other sites there were issues that limited preparedness, for example one site that had been randomized to the PDSA intervention were unable to participate in the allocated intervention because they were unable to identify a replacement facilitator after the proposed facilitator went on sick leave. Additionally sites started from a different base with their actual fasting times, ranging from one extreme of an average fluid fasting time of 12.9 hours to 5.8 hours at the other extreme (where recommendations state it is safe for patients to have clear fluids up to two hours before induction of anaesthesia). Some sites had been championing evidence-based fasting practice for some years which could explain lower baseline times. Different starting points present challenges to implementation researchers, particularly within the relative confines of a trial design and the implementation of complex interventions.

### Integration with existing initiatives

There was some evidence that aligning implementation with existing relevant activities enhanced the chances of more successful implementation. For example, in the hospital (standard dissemination) that had the largest decrease in fluid fast times (from 12.5 to 7.7 hours), they added this project to an existing initiative that had been implemented in a different department. In another site (web resource and opinion leader), they used learning and awareness from a patient safety initiative to implement individual review of fasting practice. This hospital started with the lowest pre-intervention fluid fast time (5.8 hours), which continued to get lower (4.2), but not statistically significantly lower.

In summary, the implementation context was challenging, proving resistant to the implementation interventions evaluated in this study. The behavior and choices of teams and individuals, including patients, bounded by professional issues, power and a lack of clarity for the authority and responsibility of fasting practice operated within a wider organizational system that mediated the implementation of new practices. In theory, the PDSA intervention had the potential to work with these complexities, however, the delivery of this strategy was itself compromised by the challenging environment, including lack of time and opportunity to bring people together to problem solve. However, in sites where processes were aligned with existing initiatives some progress was made.

### Facilitation

Facilitation is concerned with enabling and making things easier [30]. In PoISE the purpose of facilitation was to enable implementation through the web resource + opinion leader, and PDSA interventions. At each site there was also a key contact who facilitated the running of the project at a local level. Nurses and anaesthetists took on these roles, and in some sites this included both nurses and anaesthetists. The enactment of these roles varied and was linked to activities rather than the prescribed intervention strategy.

### Activities and impact

In interviews, opinion leaders and PDSA facilitators reported engaging in many activities including: amendment of information; dissemination of information; awareness raising; individual review of patient fast; educational meetings; policy development; promotion of guidance; teaching/training (formal and informal); and using role models of good practice.

Opinion leaders and PDSA facilitators reported operationalising these activities in the following ways:

1. Using a structure already in place to review fasting times or implement practice change, for example, adding a discussion of fasting times to pre-list theatre meetings introduced as part of The Health Foundation Safer Patient Initiative or adding some information giving process (verbal or written) to pre-assessment clinic appointments.

2. Using a real time practice opportunity to initiate practice change, for example using anaesthetic rounds to discuss fasting and educated staff in current practice.

3. Using evidence of patient discomfort to prompt practice change.

4. Using a key role, for example, theatre manager acted as liaison for reviewing fasting times for patients.

5. Putting fasting practice on the ward team’s or clinical department’s agenda to raise awareness and encourage practice change, for example, conducting an audit of ward nurses’ knowledge about fasting and current policy during shift hand over as part of a PDSA cycle to assess need for education or using a slot in educational ward or departmental meetings.

6. Starting small in targeted wards and gradually rolling out practice change.

7. Putting up posters to raise awareness of practice changes.

Given the lack of effect on duration of fasting as a primary outcome, it is difficult to judge the relative impact of the activities of opinion leaders and PDSA facilitators. However, we did record changes to policy and practice, which did not translate into changes to patient outcomes. When questioned about the relative usefulness of these activities in prompting changes, the activities that were rated higher included revised patient and staff information, feedback of audit data, working through pivotal individuals, informal teaching, and using pre-surgery safety briefings. Apart from revision of information, these activities are more active than passive.

In summary, many activities were recorded for those in facilitator roles, but their relative and direct impact on policy and practice changes were difficult to judge. There appeared to be no distinguishing features between the activities, skills, and attributes of an opinion leader and those of a PDSA facilitator in this study, despite the interventions being conceptually different, and packaged distinctively.

### Summary

Findings show that while the evidence underpinning the fasting recommendations was strong and relatively uncontested, the delivery of interventions and the practice change was mediated by many factors, including individuals’ behaviors, attitudes, emotional responses, communication by and across individuals and teams, and by challenging implementation contexts, including inter-professional functioning and an organization’s existing surgical systems and processes. Within two of the interventions there was the potential to work with individuals and teams to attempt to overcome some of the challenges, but this was not translated to reductions in fasting times in most sites. Potentially successful strategies included using existing structures or initiatives already in place to review fasting times and practice, aligning with organizational strategies, working with those in pivotal roles, and the initiation of awareness raising activities.

## Discussion

The main aim of this paper is to reflect on how findings from the process evaluation have implications for the PARIHS framework and its development as framework that represents the implementation of evidence into practice. In discussing these issues, we draw on Helfich et al.’s [17] critique of PARIHS and the three opportunities to refine the PARIHS framework they identify: being clearer about the interrelationships and dynamics between elements/sub elements would eventually help to identify more generalisable patterns; the need for a more explicit definition for successful implementation; and drawing on other conceptual frameworks and models to further elaborate on core PARIHS elements.

### Interrelationships and dynamics

Consistent with the underlying tenant of PARIHS, the findings present a multi-faceted and dynamic story of implementation. Reflecting on the proposition that successful implementation is a function of evidence, context, and facilitation, the element of evidence requires further scrutiny. Previous research indicates that where there is strong research with clinical consensus it is more likely to be used in practice [31]. In this study, the evidence for shortening fasting times is scientifically robust and was generally acceptable, but these qualities were not sufficient to outweigh other factors. Changing an organization’s systems and processes to enable individualized fasting times requires more than robust and believable evidence. In this study, the presence of good quality evidence was a constant, however it proved to be a necessary (for example, to guide the development of new local policy/guidance), but not a sufficient condition for changing current practice and routines.

Nilson et al. [32] discuss the potential role of habit theory in changing healthcare practitioners’ daily practice. They suggest that those who develop habitual behaviors are less likely to act on, or may avoid new information that challenges current practice—particularly in contexts that remain stable. These authors suggest that breaking ‘bad’ habits could be achieved by changing something in the context or by removing a person from that context. This is similar to the idea of creating a dissonance or awakening that current practice is not necessarily appropriate practice, which is a feature of other literatures including practice development [33]. In this study, the information in recommendations would not have been new to most practitioners; however, one explanation is that their practices were habituated towards maintaining traditional (non evidence-informed) ways of working, which then becomes their stable (and familiar) context. Equally, it could be suggested that organizations and systems become habituated, such that in the case of fasting for example, 12-hour fasts become so embedded and institutionalized that this standard becomes the acceptable norm. The link between behavior and context is made explicit by habit theory, and fits well with the relationship between evidence and context in PARIHS.

Helrich et al. [17] suggest that the high-low continua within PARIHS could encourage a tendency towards linear relationships between elements. In this study, we had difficulty mapping the findings onto the high-low continua, which could be illustrative of two things. First, that the ideal position of the elements may vary from project to project, such that in some initiatives for example, it is not always necessary to have ‘high’ evidence and ‘high’ context alongside ‘high’ (appropriate) facilitation, which is the current theory of PARIHS. Second, because each implementation project will have a particular dynamic and multiple interconnections that may vary throughout its lifetime, it is not possible to plot this on a high-low continuum. Using the high-low continuum may be more helpful in providing a visual representation at diagnosis (i.e., a snapshot), but less useful in evaluating the process of implementation because this does not capture dynamism and patterns of interactions over time.

Findings from this study show that some factors were more important than others in providing the conditions for, and influencing the effect of implementation interventions, which included inter-professional team working (including communication), decision-making authority (mediated by inter-professional tensions), and organizational buy-in, issues evident in others’ work [e.g., [34,35]. The interventions, including facilitation components, did not overcome the challenges presented by these factors to a sufficient level to affect fasting outcomes even though facilitators reported working with individuals and teams on a variety of activities. Therefore the main interactions in this study were between individuals and teams and context. Currently individuals are not explicitly part of the PARIHS framework but are embedded implicitly within evidence (individuals interact with evidence), context (individuals are part of context), and facilitation (facilitators work with individuals and teams). A case for making individuals more explicit within PARIHS is made below.

### Successful implementation

Successful Implementation (SI) has not been explicitly defined in previous PARIHS publications. Helfrich et al. [17] suggest that successful implementation should take a logic model approach to linking the implementation strategy to outcomes, including the realization of an implementation plan, the achievement and maintenance of the targeted evidence-based practice, and the achievement and maintenance of patient or organizational outcomes. A logic model can (although does not have to) encourage a linear and deductive approach to the identification of inputs, processes and outcomes, which does not fit well with the underlying premise of PARIHS, which acknowledges dynamism and the potential for inductive explanation [36]. For this project, successful implementation was defined in broad terms as the use of the recommendations in practice with associated impact on practice and patient outcomes. A more helpful definition, which acknowledges implementation as a process might be: an orchestrated (active, planned) effort to make evidence-based changes by organizations, teams, and individuals that result in sustained improvements to care, patient outcomes, and service delivery, which are driven by and embedded in organizational strategy. This definition includes the need to pay attention to planning, the process, and evaluation of implementation activity in an iterative rather than staged approach. It is a definition that could apply equally to one-off implementation projects, such as this guideline implementation study, as to initiatives or programs that intend to create the conditions for sustained use of evidence and improvements in practice, such as the Collaborations for Leadership in Applied Health Research and Care in England and the Department for Veterans Affairs in the United States.

### Elaborating on PARIHS elements

Helfrich et al. encourage better elaboration of PARIHS’ core elements. To date, the role, behavior and attributes of individuals have been implicit within the PARIHS framework. Findings from this study and evidence from others’ research and conceptualisations of evidence-based change and theory show the crucial role that individuals plays in the relative success of evidence-based change [6,7,27,37-39]. Over the last decade or so there has been a shift away from a focus on individuals (in the context of the evidence-based practice movements) to one that recognizes the role that context plays in implementation. Arguably this shift has resulted in an inattention to the study of individual factors, specifically the interplay between actors and the contexts in which they work and how that interplay influences change processes and impacts. Evidence suggests that many individual level factors including beliefs, attitudes, motivations, values, skills, competence, behavior, and characteristics may be influential [40,41]. These are consistent with findings in the current study where individual’s risk taking behavior, emotional response, skills and experience, enthusiasm, commitment, and decision-making authority were important factors in the intervention’s implementation and impact.

This is a timely opportunity to consider the inclusion of individuals as an explicit additional element to PARIHS. Individuals are currently implicitly embedded within PARIHS in that facilitators work with individuals, contexts include individuals, and individuals interact with evidence, however, the significance of individuals within implementation is perhaps currently under-represented. In other theories (e.g., Rogers Diffusions) and frameworks (e.g., [6,8,27], the individual is acknowledged as a core component. Therefore we suggest that individuals should be represented explicitly in the PARIHS framework so that successful implementation related to how individuals (at an individual, team, and organization level) interact with evidence, context, and how these interactions are facilitated towards successful processes and outcomes. Our findings do not suggest that evidential factors be displaced by individual factors; rather, the observable pattern of interaction between evidence, context, and facilitation was influenced by individual (patients and practitioners) and collective behavior such as over-estimating risk, caution, and team functioning. Therefore, it could be argued that individual’s behavior, intentions and actions should be part of a framework that seeks to explain successful implementation.

A new representation of the PARIHS framework, including an in-depth consideration of the implications of including a new component related to individuals’ or actors’ characteristics, behavior, actions, and how this impacts on the development of this middle range theory, will be the subject of a future publication. However, drawing on the components of other frameworks, theories, and evidence [e.g., [6-8,27,37,40-42], we propose that the concept of the individual might incorporate: capability, capacity, motivation (including recognising a need for change), resilience, acceptability, feelings, knowledge and beliefs (including self efficacy) about the intervention/evidence, position and fit within the organization/social system, and approach to decision-making (e.g., experimentation, use of information). It is important to state that making individuals more explicit within the PARIHS framework does not mean they should be separated from the other elements, particularly context. The strength of the framework is in representing the elements’ interconnectivity, which should be preserved in this new representation. As such, this addition could be summarized in the following updated working proposition:

The successful implementation of evidence into practice is a planned facilitated process involving an interplay between individuals, evidence, and context to promote evidence-informed practice.

The additional element has the potential to strengthen the framework’s usefulness for planning and evaluating implementation efforts and is a reflection of current evidence and theory, particularly discussions about the social processes involved in knowledge mobilisation work [43].

### Limitations

The process evaluation was designed to capture data across intervention sites, rather than conduct in-depth case studies or ethnographies in a few sites. In-depth data collection within purposively sampled sites may have provided more illuminating evidence about intervention implementation, particularly fidelity. We also did not have the capacity to undertake any observational work, and therefore have been reliant on self-reported data to reach the conclusions reported in this paper. Additionally, there are some voices missing from this account, including those of surgeons (who could have participated, but did not consent to) and senior operational managers (who were not included in our sampling strategy).

Although the study design and interventions were prospectively designed based on the core elements of PARIHS, our evaluation of the framework has been retrospective. The addition of the concept of the individual needs further consideration, elaboration and clarification.

## Conclusions

The process evaluation reported here was embedded in one of the first and largest implementation randomized controlled trials within acute care. The findings from this evaluation lead us to proposing an extension to the PARIHS framework to enhance its usefulness as a conceptual framework that can be applied in practice. To date, this framework has received much attention from the international implementation community as a helpful representation of the ingredients for successful implementation. The framework has been developed and refined over time, therefore the addition of ‘individuals’ to the conceptualisation of successful implementation is timely, particularly in the context of a growing emphasis in the empirical and theoretical literature on behavior and social processes. The findings from this study re-emphasize the multiple and complex deliberative processes involved in implementation work. In this paper, we propose that the PARIHS framework should be enhanced to ensure theoretical development keeps a pace with the current evidence base for implementation.

## Competing interests

The authors declare they have no competing interests.

## Authors’ contributions

JRM conceived the study, and led the design in collaboration with KS, IB, and NC. JRM, KS, and IB secured funding. JRM supervised all aspects of the study, with input from KS. JC and CH coordinated and took the lead role in data collection and analysis. CA provided a patient perspective throughout the conduct of the study including the development of patient related materials and data collection processes. LS provided a clinical perspective as an aneasthetist. JRM drafted the paper KS provided feedback on early drafts, NC, CH, JC, CA, IB reviewed later drafts for intellectual comment and all approved the final manuscript.
